# Erianin promotes apoptosis and inhibits Akt-mediated aerobic glycolysis of cancer cells

**DOI:** 10.7150/jca.92780

**Published:** 2024-03-04

**Authors:** Shuangze Han, Sijin Chen, Jidong Wang, Sheng Huang, Yeqing Xiao, Gaoyan Deng

**Affiliations:** 1 Department of Thoracic Surgery, Hunan Chest Hospital, Changsha 410013, Hunan, China.; 2 Department of Ultrasound, Union Hospital, Tongji Medical College, Huazhong University of Science and Technology, Wuhan 430022, China.; 3 Department of Urology, Hunan Provincial People's Hospital, The First Affiliated Hospital of Hunan Normal University, Changsha 410005, Hunan, China.; 4 Department of Oral and Maxillofacial Surgery, Changde Hospital, Xiangya School of Medicine, Central South University (The first people's hospital of Changde city), Changde 415000, Hunan, China.; 5 Department of General, Hunan Chest Hospital, Changsha 410013, Hunan, China.; 6 Department of Ultrasonography, Hunan Chest Hospital, Changsha 410013, Hunan, China.

**Keywords:** erianin, aerobic glycolysis, HK2, non-small cell lung cancer

## Abstract

Highly activated aerobic glycolysis provides the metabolic requirements for tumor cell growth and proliferation. Erianin, a natural product isolated from *Dendrobium chrysotoxum Lindl*, has been reported to exert antitumor activity in multiple cancers. However, whether Erianin exerts inhibitory effects on aerobic glycolysis and the inherent mechanism remain poorly defined in non-small cell lung cancer (NSCLC). Here, we showed that Erianin inhibited the cell viability and proliferation, and induced apoptosis in NSCLC cells. Moreover, Erianin overtly suppressed aerobic glycolysis via decreasing HK2 expression. Mechanistically, Erianin dose-dependently curbed the Akt-GSK3β signaling pathway phosphorylation activation, which afterwards downregulated HK2 expression. Meanwhile, Erianin inhibited HCC827 tumor growth *in vivo.* Taken together, our results suggest that the natural product Erianin can suppress aerobic glycolysis and exert potent anticancer effects via the Akt-GSK3β signaling pathway in NSCLC cells.

## Introduction

Lung cancer is the most common cause of cancer-related deaths worldwide, among which non-small cell lung cancer (NSCLC) accounts for 80%-85%^1^-^4^. Over the past decade, the treatment paradigm for NSCLC has progressed dramatically including chemotherapeutic agents, molecularly targeted drugs, and immune checkpoints inhibitors^5^-^12^. However, the vast majority of advanced NSCLC become resistant to current treatments and eventually progress^1^, ^13^, ^14^. Therefore, the clinical treatment for NSCLC urgently demands identifying novel targets and elucidating underlying mechanisms.

Erianin is a natural product extracted from *Dendrobium chrysotoxum Lindl*^15^, ^16^. Increasing evidence has manifested that Erianin exerts antitumor properties through diverse molecular mechanisms^16^, ^17^. Erianin induces apoptosis, blocks angiogenesis, and inhibits cancer progression through the MAPK^18^, and mTOR pathways^19^, ^20^. In lung cancer, Erianin inhibits cell growth and migration via PI3K/Akt pathway^21^, and calcium/calmodulin-dependent ferroptosis^22^, ^23^. Erianin suppresses lung cancer stemness via ferroptosis^24^. These studies imply that Erianin is a potent and promising antitumor agent for clinical treatment.

However, the impact of Erianin on aerobic glycolysis in lung cancer cells remains unclear. In this study, we revealed the inhibitory effects of Erianin on NSCLC cells and found that Erianin significantly suppressed aerobic glycolysis in NSCLC cells. Herein, we further unveiled the underlying mechanisms of Erianin administration against NSCLC cells.

## Materials and methods

### Cell culture and antibodies

Human NSCLC cell HCC827, HCC1975, H1650, and H460 cells were obtained from American Type Culture Collection (ATCC, Manassas, VA). All cells were cultured in DMEM medium supplemented with 10% Fetal Bovine Serum (FBS) and 1% penicillin-streptomycin at 37°C with 5% CO_2_. All cells were maintained at the incubator according to the standard protocols and subjected to routinely checking for mycoplasma contamination. Antibodies against HK2 (#2867), Akt (#4691), p-Akt (#4060), cleaved-caspase 3 (#9664), Bax (#14796), VDAC1 (#4661), α-tubulin (#2144), cytochrome c (#4280), Akt1 (#2938), Akt2 (#2964), p-GSK3β (#5558), β-actin (#3700), anti-rabbit IgG HRP (#7074), and anti-mouse IgG HRP (#7076) were obtained from Cell Signaling Technology, Inc. (Beverly, MA). The natural compound Erianin was from Selleck Chemicals (Houston, TX). Necrostatin-1, z-VAD-fmk, and 3-methyladenine were purchased from MedChemExpress (New Jersey, US). Lipofectamine 2000 transfection reagent for transient transfection was purchased from Thermo Fisher Scientific (Waltham, MA).

### MTS assay

MTS assay was performed according to the standard protocol^25^. The cultured cells were resuspended and seeded into 96-well plates (8x10^3^ cells/well) and were incubated at different time points. Cell viability was analyzed with MTS using the Cell Titer 96^®^ Aqueous One Solution kit (Promega Corporation) as determined by the manufacturer's protocol.

### Soft Agar Assay

The soft agar assay was performed as described previously^26^. Briefly, the agar base was made with 3 mL of Eagle's basal medium supplemented with 0.6% agar and 10% FBS in a 6-well plate. Cells were collected and counted at 8x10^3^ cells/mL concentration in 1 mL of Eagle's basal medium supplemented with 0.3% agar and 10% FBS overlaid into a 6-well plate with 0.6% agar base. The cells were routinely cultured for 14 days. The colony number was counted with the microscope.

### Plate colony formation assay

The cultured cells were exposed to Erianin and were routinely incubated for 2 weeks in a 6-well plate (500 cells/well). When cells formed sufficiently large colonies, cells were fixed with 4% paraformaldehyde for 20 min at 37 ˚C. Cells were stained with 0.5% crystal violet for 5 min at 37 ˚C. The number of colonies was counted with a microscope.

### Glycolysis analysis

The glycolysis analysis was performed as described previously^27^. Glucose consumption and lactate production were analyzed at the Laboratory the Third Xiangya Hospital of Central South University (Changsha, China). The relative glucose consumption and lactate production rate were normalized by protein concentration.

### Subcellular Fraction Isolation

The Mitochondria Isolation kit (Thermo Fisher Scientific, Inc.) was used for cytosolic and mitochondrial fraction extraction following the manufacturers' instructions.

### Cell Transfection

For transient transfection, the siRNAs, including si-Akt (sc-29195) and siCtrl (sc-37001), were purchased from Santa Cruz Biotechnology (Dallas, TX). The transient transfection was performed using the Lipofectamine 2000 (11668019, Thermo Fisher Scientific) following the manufacturer's protocol. The whole cell extract was prepared at 2 days later after transfection.

### Immunoblotting

The immunoblotting (IB) was performed as described previously^28^. Briefly, Cells were lysed in RIPA lysis buffer (Thermo Fisher Scientific, Inc.) containing protease inhibitors to obtain whole-cell extract (WCE), whose concentration was determined by BCA protein assay (Thermo Fisher Scientific, Inc.). Equal amounts of protein (30 μg) were mixed with loading buffer, boiled at 95°C for 5 min, then subjected to SDS-PAGE electrophoresis and transferred onto a PVDF membrane. The membranes were incubated with the primary antibody overnight at 4°C after blocking with 5% non-fat milk at room temperature (RT) for 1 h. Finally, the secondary antibody anti-rabbit/mouse IgG HRP was added and incubated for 30 min at RT, and then the target protein was visualized by chemiluminescence.

### Immunofluorescence (IF)

The IF analysis was performed as described previously^29^. Briefly, Cells were fixed in 4% paraformaldehyde (sc-281692; Santa Cruz Biotechnology, Inc.) for 10 min, and permeabilized in 0.2% Triton X-100 (13444259; Thermo Fisher Scientific, Inc.) for 20 min. The slides were incubated with antibodies overnight at 4°C in a humidified chamber after blocking with 50% goat serum albumin. The next day, the fluorescence-labeled second antibody was added for 40 min at RT. DAPI was used for counterstaining. The stained cell images were obtained using the fluorescence microscope.

### Xenograft mouse model

All *in vivo* animal experiments were approved by the Institutional Animal Care and Use Committee (IACUC) of Central South University (Changsha, China). HCC827 cells (2 × 10^6^) in 200 μl DMEM were harvested and subcutaneously inoculated in the right flank of 6-week-old athymic nude mice (n = 6) to generate xenograft models. The tumor volume and body weight of mice were recorded every 2 days. The tumor-bearing mice were randomly divided into two groups when the tumor reached ~100 mm^3^. The compound-treated group was administrated Erianin (10 mg/kg every 2 days) by intraperitoneal injection, whereas the control group was administrated the vehicle control. Tumor volume was calculated as length × width^2^ × 0.5. The mice were euthanized at the endpoint, and the tumor tissues were dissected.

### Blood assay

Blood was collected via cardiac puncture of mice with EDTA coating pipes. The red blood cells (RBC), white blood cells (WBC), alanine aminotransferase (ALT), and aspartate aminotransferase (AST) were examined in the laboratory of Central South University of China.

### Immunohistochemical staining (IHC)

The IHC staining was performed as described previously^25^. Briefly, the tissues of xenograft tumors were fixed in 10% neutral-buffered formalin. The tissue sections were processed to repair the antigen by the following steps: deparaffinizing, rehydrating, submerging in sodium citrate buffer (10 mM, pH 6.0), and boiling for 10 min. The tissue slides were next treated with 3% H_2_O_2_ at room temperature for 10 min, washed with PBS, and blocked with 10% goat serum albumin, followed by incubating with primary antibodies overnight at 4 ℃ and secondary antibodies at room temperature for 45 min. The interested protein was visualized through the DAB substrate incubation and counterstained by hematoxylin. The scores for IHC staining were determined and analyzed by two senior pathologists with a microscope and the Image-Pro Plus software (version 6.2) program (Media Cybernetics). To obtain the scores of the targeted protein we used the calculation formula: percentage scores × intensity scores. The percentage of positive cells was classified into four categories: 0, no positive cells; 1, ≤ 10% positive cells; 2, 10-50% positive cells; 3, > 50% positive cells. The intensity was graded as 0, no staining; 1, weak staining; 2, moderate staining; 3, intense staining. The IHC scores of Ki67, HK2 and p-Akt expression were interpreted as follows: ≤ 2 indicates low expression level; > 2 indicates high expression level.

### Statistical analysis

SPSS software (version 13.0; SPSS, Inc.) was used for statistical analysis. The data are presented as the mean ± SD and were analyzed using the Student's t-test or ANOVA. P<0.05 was considered to indicate a statistically significant difference.

## Results

### Erianin inhibits the cell viability and proliferation of NSCLC cells

**Figure 1:**
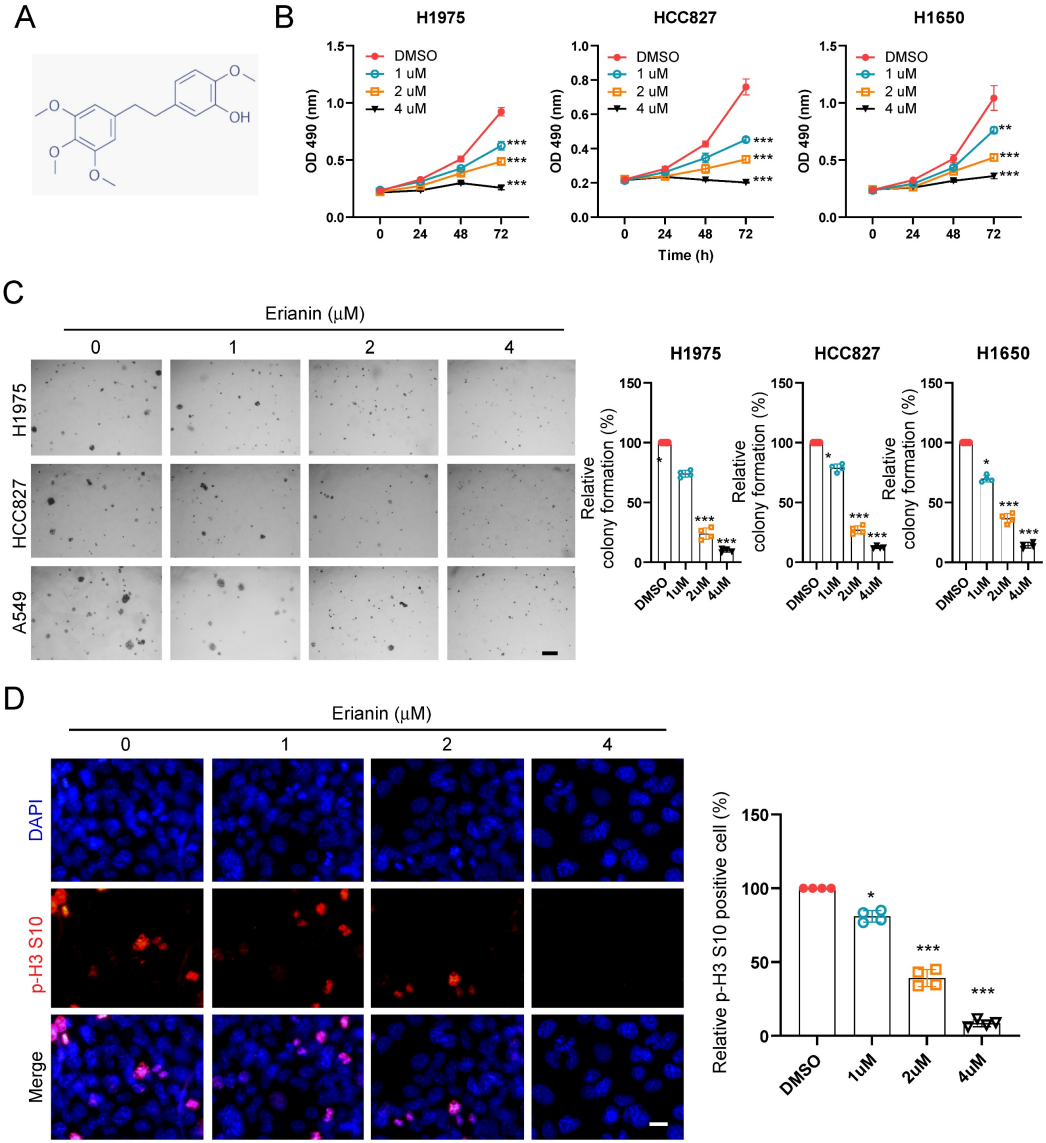
Erianin suppresses cell viability and colony formation of NSCLC cells. (a) Chemical structure of Erianin. (b) H1975, HCC827, and H1650 cells were treated with different concentrations of Erianin (0, 1, 2, 4 μM) for the indicated times. Cell viability was measured using an MTS assay. (c) Colony formation of H1975, HCC827, and H1650 cells were exposed to different concentrations of Erianin (0, 1, 2, 4 μM), Scale bar, 500 μm. (d) HCC827 cells were subjected to Immunofluorescence (IF) analysis with the histone H3 S10 antibody, Scale bar, 10 μm. *, *p* < 0.05, **, *p* < 0.01, ***, *p* < 0.001.

To determine the inhibitory effect of Erianin on NSCLC cells, we first detected the cell viability of NSCLC cells at indicated time points after exposure to different concentrations of Erianin (Figure 1A). The MTS data indicated that Erianin significantly reduced cell viability in H1975, HCC827, H1650, and H460 cells (Figure 1B and Supplementary Figure 1A). Furthermore, the colony formation ability of NSCLC cells was examined. The results indicated that the colony numbers were dose-dependently reduced with Erianin treatment in H1975, HCC827, H1650, and H460 cells (Figure 1C and Supplementary Figure 1C). Consistently, the immunofluorescence result indicated that higher concentration of Erianin significantly decreased the population of histone H3 Ser10 positive cells (Figure 1D). In contrast, the administration of Erianin had no significant effect on cell viability and colony formation in immortalized non-tumor lung cells NL20 and MRC5 (Supplementary Figure 1B and 1D). Overall, these results suggested that Erianin attenuated the cell viability and proliferation of NSCLC cells.

### Erianin suppressed aerobic glycolysis via downregulating HK2 expression in NSCLC cells

**Figure 2:**
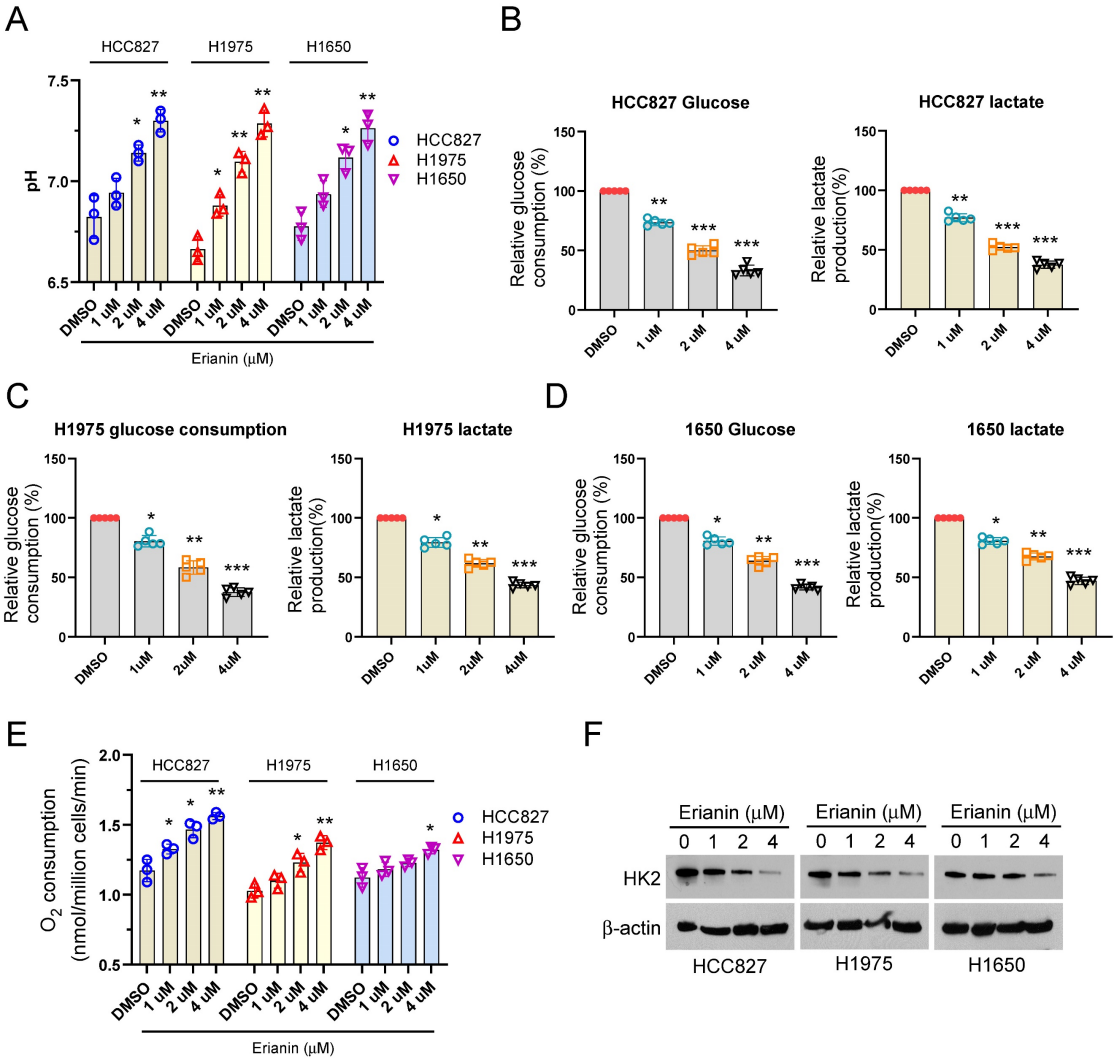
Erianin suppresses HK2 expression and aerobic glycolysis in NSCLC cells. (a-e) Normalized pH value (a), glucose consumption, lactate production, and O_2_ consumption (e) in HCC827 (b), H1975 (c), and H1650 (d) cells exposed to different concentrations of Erianin (0, 1, 2, 4 μM), respectively. (f) IB analysis of HK2 in HCC827, H1975, and H1650 cells exposed to different concentrations of Erianin (0, 1, 2, 4 μM). *, *p* < 0.05, **, *p* < 0.01, ***, *p* < 0.001.

Clinically, our analysis results illustrated that high HK2 expression had the worse OS and PFS than low HK2 expression (Supplementary Figure 2A-B). To further determine the effect of Erianin on aerobic glycolysis of NSCLC cells. Exposed to different concentrations of Erianin, the glucose metabolic characteristic was examined among different NSCLC cells. Our data showed that Erianin dose-dependently increased the pH value in H827, HCC1975, and H1650 cells (Figure 2A). Moreover, Erianin significantly dose-dependently decreased the glucose consumption, lactate production, and increased the O_2_ consumption ratio in different NSCLC cells, respectively (Figure 2B-E). Hexokinase 2 (HK2), the first-rate limiting enzyme of glucose metabolism, is required for aerobic glycolysis to phosphorylate glucose. Therefore, we further investigated the effect of Erianin on HK2, the IB results showed that Erianin dose-dependently reduced the expression of HK2 protein in HCC827, HCC1975, and H1650 cells (Figure 2F). These results suggested that Erianin suppressed aerobic glycolysis in a HK2-dependent manner in NSCLC cells.

### Erianin promotes apoptosis to inhibit the cell viability of NSCLC cells

**Figure 3:**
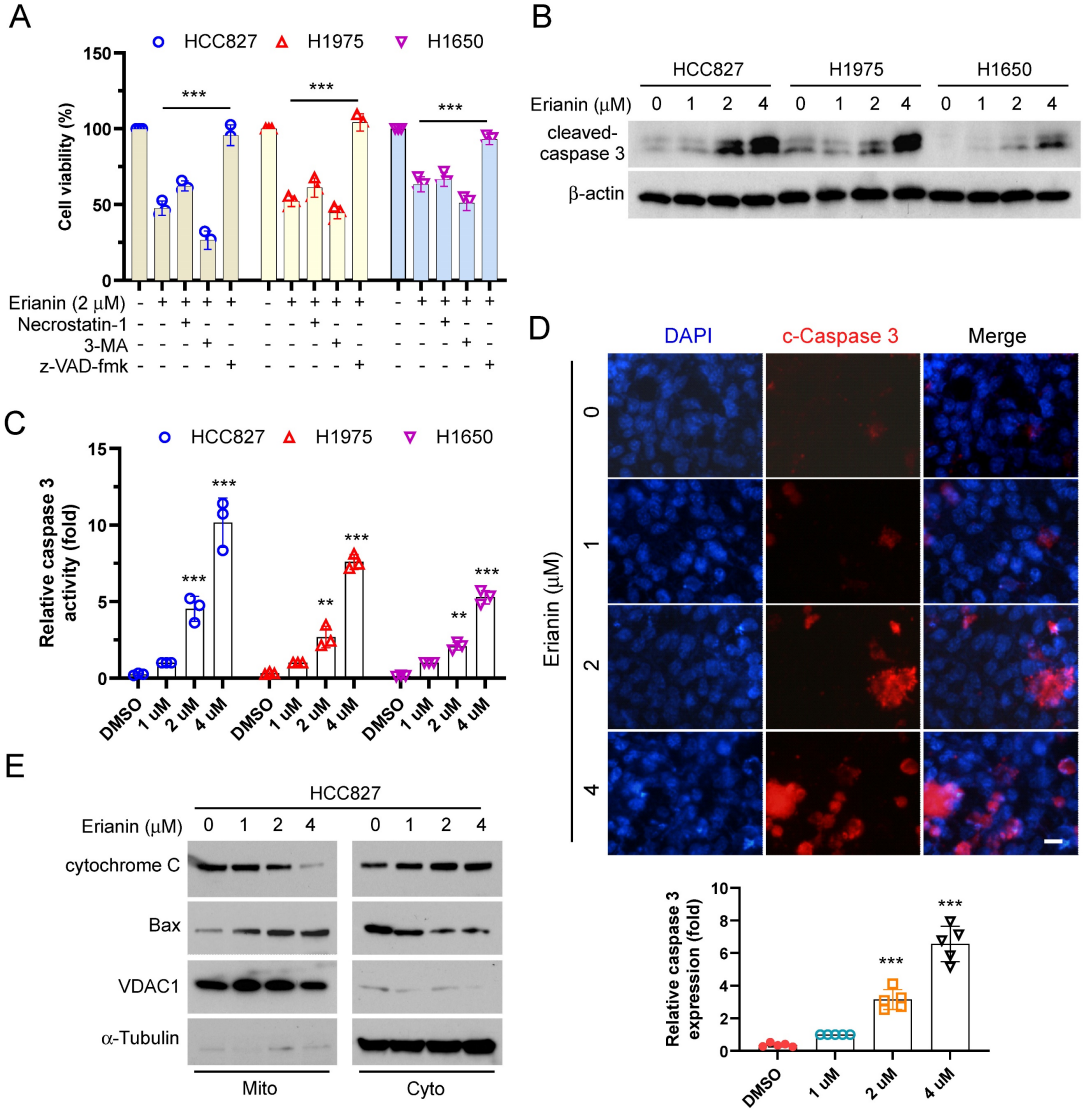
Erianin promotes apoptosis of NSCLC cells. (a) HCC827, H1975, and H1650 cells were treated with 2 μM Erianin combined with Necrostatin-1, z-VAD-fmk, and 3-MA. Cell viability was examined using MTS assay. (b and c) HCC827, H1975, and H1650 cells were treated with different concentrations of Erianin (0, 1, 2, 4 μM) for 24 h. The cell lysate was subjected to IB analysis. (d) HCC827 cells were subjected to Immunofluorescence (IF) analysis with the cleaved-caspase 3 antibody, Scale bar, 10 μm. (e) HCC827 cells were treated with different concentrations of Erianin (0, 1, 2, 4 μM) for 48 h, subcellular fractions were isolated and subjected to IB analysis. *, *p* < 0.05, **, *p* < 0.01, ***, *p* < 0.001. IB, immunoblotting.

To elucidate the inherent mechanism that Erianin inhibited the cell viability of NSCLC cells. We utilized three cell death inhibitors that inhibited necroptosis, autophagy, and apoptosis, respectively. As shown in Figure 3A, treated with Erianin and the pan-caspase inhibitor z-VAD-fmk, the cell viability was recovered significantly. The results suggested that inhibition of apoptosis rescued Erianin-induced inhibitory effects on NSCLC cells. The IB data showed that Erianin dose-dependently incremented the protein level of cleaved-caspase 3 in HCC827, HCC1975, and H1650 cells (Figure 3B-C). Moreover, the immunofluorescence indicated that Erianin increased the expression of cleaved-caspase 3 in HCC827 cells dose-dependently (Figure 3D). In addition, IB analysis further indicated that Erianin promoted the release of cytochrome c from the mitochondria to the cytoplasm and dose-dependently augmented the expression of Bax in the mitochondrial fraction (Figure 3E), suggesting that Erianin could activate the intrinsic apoptotic pathway. These results indicated that Erianin induced apoptosis in NSCLC cells.

### Akt is required for Erianin-induced aerobic glycolysis inhibition of NSCLC cells

**Figure 4:**
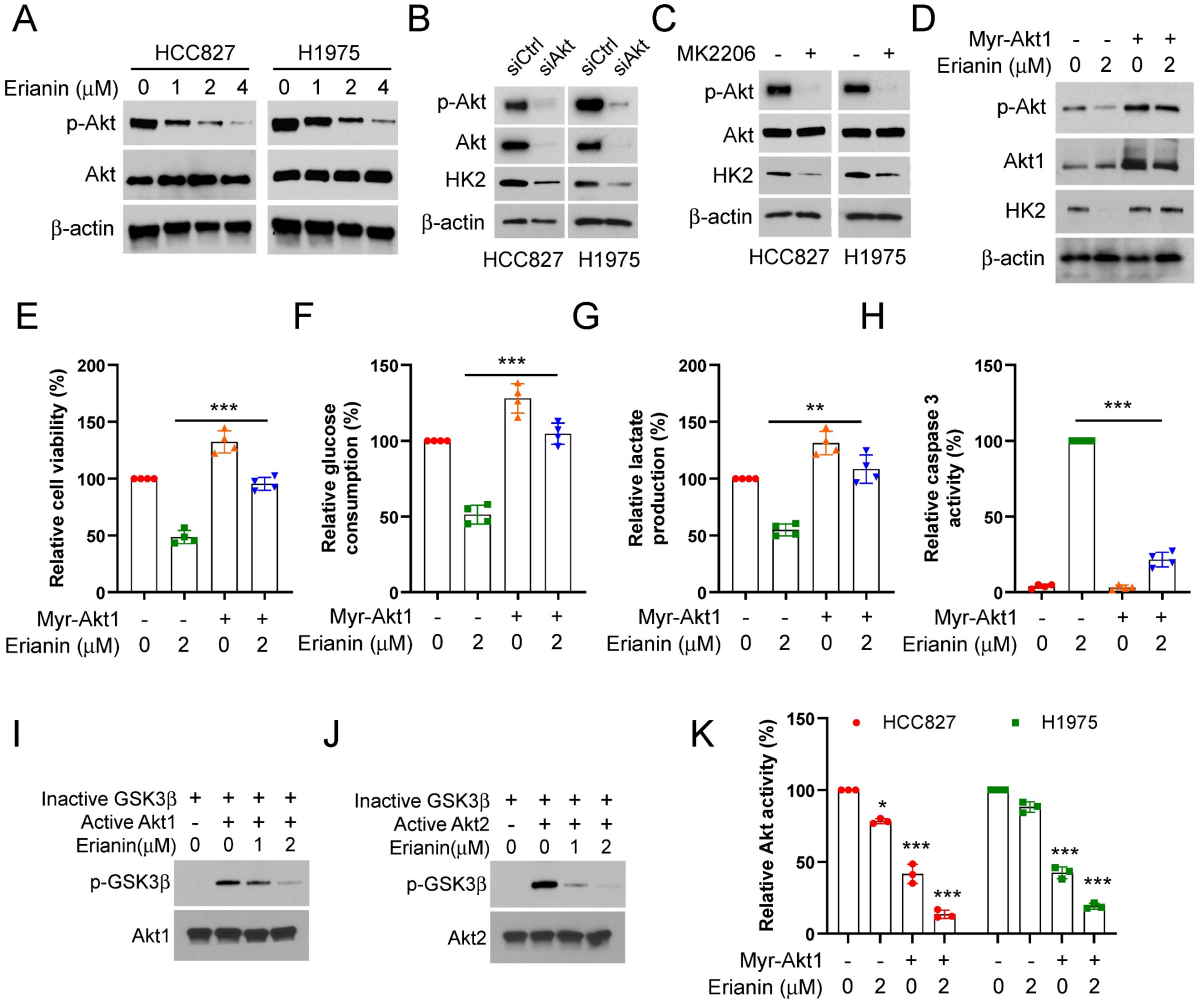
Erianin induces Akt inactivation to inhibit aerobic glycolysis of NSCLC cells. (a) HCC827 and H1975 cells were treated with various concentrations of Erianin, the WCE was subjected to IB assay. (b) HCC827 and H1975 cells were transfected with siCtrl or siAkt for 24h, followed by IB assay. (c) HCC827 and H1975 cells were treated with MK2206 for 24h, followed by IB assay. (d-h) Myr-Akt1 was transfected into HCC827 cells for 24h, followed by 2 μM Erianin treatment. The cell lysate was subjected to IB analysis (d). MTS analysis of cell viability (e). The glucose consumption (f) and lactate production (g) were detected. Caspase 3 activity was determined using the Caspase 3 activity assay (h). (i-j) *In vitro* kinase assay analysis of the inhibitory effect of Erianin on Akt1 (i) and Akt2 (j). (k) Myr-Akt1 was transfected into HCC827 and H1975 cells, followed by Erianin treatment. Akt activity was measured by Akt Kinase Activity Assay Kit. **, *p* < 0.01, ***, *p* < 0.001.

Our study further determined the inhibitory mechanism of Erianin on aerobic glycolysis in NSCLC cells. We found that Erianin dose-dependently suppressed the phosphorylation of Akt (Figure 4A). Meanwhile, IB analysis indicated that Akt knockdown could decrease the protein level of HK2 (Figure 4B). Likewise, treatment with the kinase inhibitor MK2206, which can specifically reduce the activity of Akt kinase, caused a robust decrease of HK2 protein in HCC827 and HCC1975 cells (Figure 4C). Conversely, in the presence of Erianin, ectopic expression of constitutively activated Akt1 (Myr-Akt1) restored Akt phosphorylation and consistently increased the level of HK2 and the cell viability (Figure 4D-E). The glycolysis analysis results further confirmed that overexpression of Myr-Akt1 enhanced glucose consumption and lactate production (Figure 4F-G). However, the activity of caspase 3 was obviously inhibited (Figure 4H). Meanwhile, Erianin dose-dependently suppressed the phosphorylation level of GSK3β (Figure 4I-J), which attributed to Erianin-mediated upstream Akt kinase activity inhibition (Figure 4K). Our results illuminated that Erianin inactivated the Akt-GSK3β signaling pathway, and Akt was required for the Erianin-mediated inhibition of aerobic glycolysis on NSCLC cells.

### Erianin suppresses tumor growth of NSCLC cells *in vivo*

**Figure 5:**
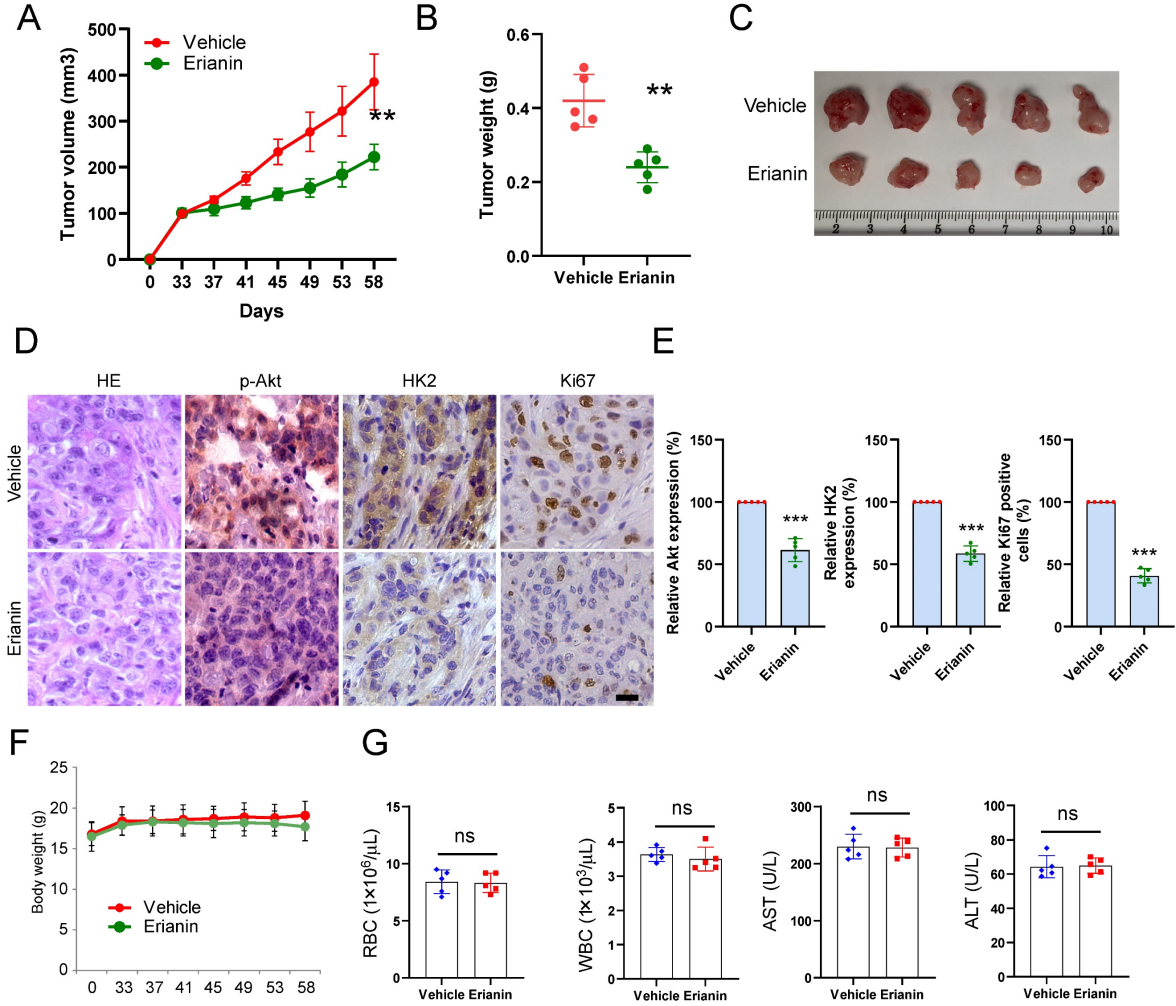
Erianin inhibits *in vivo* tumor growth of NSCLC cells. (a-c) The tumor volume (a), tumor weight (b), and the image of tumor mass (c) of HCC827-derived xenograft tumors treated with vehicle and Erianin. (d-e) The representative images (d) and qualifications (e) of IHC staining of p-Akt, HK2, and Ki67 in HCC827-derived xenograft tumors with Erianin or vehicle treatment. Body weight (f) and mouse blood assay (g) after treatment with erianin or vehicle. Scale bar, 20 μm. **, *p* < 0.01, ***, *p* < 0.001, ns, no significance.

The xenograft mouse model was constructed to verify the inhibitory effect of Erianin on tumor growth *in vivo*. HCC827-deprived xenograft tumors were exposed to Erianin. The results illustrated that Erianin significantly inhibited the growth of HCC827 tumors compared with the vehicle control (Figure 5A and Figure 5C). The tumor weight of the Erianin-treated group was lower than that of the vehicle-treated group (Figures 5B). Moreover, IHC staining showed that Erianin reduced the expression of phosphorylated Akt and HK2 and decreased the population of Ki-67 positive cells (Figure 5D-E). In addition, the body weight did not change significantly between the Erianin- and vehicle-treated groups (Figure 5F). Erianin treatment did not exhibit specific toxicity on any critical organ functions. When compared to the vehicle-treated group, the levels of red blood cells (RBC), white blood cells (WBC), aspartate transaminase (AST), and alanine transaminase (ALT) were unaffected by Erianin administration (Figure 5G). Overall, these results indicated that the administration of Erianin effectively suppressed tumor growth of NSCLC cells *in vivo*.

## Discussion

Non-small cell lung cancer (NSCLC) is a profoundly devastating disease that is the leading cause of cancer-associated deaths worldwide^30^-^33^. NSCLC accounts for more than 85% of lung cancer cases, of which lung adenocarcinoma (LUAD) and lung squamous cell carcinoma (LUSC) are the most common subtypes^34^-^37^. Over the past decade, significant advances have been made in non-small cell lung cancer therapies, Nevertheless, the incidence and mortality have remained increasing, with the predicted 5-year survival rate of 15.9%^38^-^43^. Aerobic glycolysis, referred to as the Warburg effect, provides the metabolic requirements for tumor cell growth and proliferation^44^-^47^. It is characterized by increased glucose uptake and lactate production^48^-^51^. Unlike normal cells that catabolize glucose by oxidative phosphorylation in the mitochondria, tumor cells tend to convert glucose into lactate regardless of oxygen availability^52^-^55^. Meanwhile, the metabolic intermediates generated during aerobic glycolysis can be used for the biosynthesis of biomacromolecules used by the tumor to meet the demands for rapid growth^56^-^60^. The lactate production also provides an acidic environment to aid the invasion and metastasis of cancer^61^-^63^. The first step of glycolysis is catalyzed by hexokinase (HK), which converts glucose to glucose-6-phosphate (G-6-P)^52^, ^64^, ^65^. Among four HK isoforms, HK2 is overexpressed in many cancer cells. Han et al.^66^ found that the deubiquitinase OTUB1 stabilized MYC and induced HK2 expression, which leads to the promotion of aerobic glycolysis and breast tumorigenesis. In hepatocellular carcinoma, UBR7 inhibits glycolysis by indirectly suppressing HK2 expression^67^. Thus, we attach much importance to the role of HK2-mediated aerobic glycolysis in NSCLC.

Erianin, a natural product derived from Dendrobium chrysotoxum Lindl^23^, has extensive and potent pharmacological activities, including anti-tumor^68^, anti-inflammatory^69^, and anti-bacterial^20^. Previous studies have demonstrated that Erianin exerted strongly effective anti-tumor effect by promoting apoptosis^21^, ferroptosis^70^, and autophagy^71^, and inhibiting angiogenesis^72^. However, the role of Erianin on aerobic glycolysis in NSCLC remains unclear. In this research, we observed the effect of Erianin on NSCLC cells. Erianin promoted apoptosis and subsequently attenuated the cell viability and proliferation in a dose-dependent manner (Figure 1B-D). Furthermore, our data indicated that Erianin exerted inhibitory effects via suppressing HK2-mediated aerobic glycolysis in NSCLC cells. Erianin significantly dose-dependently decreased glucose consumption and lactate production and reduced the expression of HK2 protein (Figure 2B-F). The results suggested the indispensable role of HK2 in aerobic glycolysis. The inherent mechanism study revealed that Erianin suppressed the phosphorylation of Akt kinase. Akt knockssdown and inhibition could robustly suppress HK2-mediated aerobic glycolysis (Figure 4A-B). However, overexpression of Myr-Akt1 enhanced the glucose consumption and lactate production (Figure 4F-G). Meanwhile, as the downstream target of Akt kinase, Erianin dose-dependently suppressed the phosphorylation level of GSK3β (Figure 4I-K). Therefore, our results illuminated that Erianin inactivated the Akt-GSK3β signaling pathway to inhibit aerobic glycolysis in NSCLC cells.

In summary, our research demonstrated that Erianin was a new aerobic glycolysis inhibitor in NSCLC cells. Our data will hopefully support the use of Erianin as a potential compound for NSCLC treatment *in vitro* and maximize the potential of Erianin for application in the future.

## Supplementary Material

Supplementary figures.

## Abbreviations

3-MA: 3-methyladenine
MAPK: mitogen-activated protein kinase
mTOR: mechanistic target of rapamycin
PI3K: phosphoinositide 3-kinase
Akt: protein kinase B
GSK3β: glycogen synthase kinase-3β
HK2: hexokinase 2
IB: immunoblotting
IF: immunofluorescence
OS: overall survival
PFS: progression-free survival
