# Supplementary material for: Erianin promotes apoptosis and inhibits Akt-mediated aerobic glycolysis of cancer cells
Source: J Cancer. 2024 Mar 4;15(8):2380–90. doi: 10.7150/jca.92780 (PMC10937289; doi:10.7150/jca.92780)

Supplementary Figure 1. The effect of Erianin on NSCLC cells H460 cells and normal lung cells NL20 and MRC5. (a-b) H460, NL20 and MRC5 cells were treated with different concentrations of Erianin (0, 1, 2, 4 μM) for the indicated times. Cell viability was measured using an MTS assay. (c-d) Colony formation of H460, NL20 and MRC5 cells were exposed to different concentrations of Erianin (0, 1, 2, 4 μM), Scale bar, 500 μm. * *, $p < 0.01$, ***, $p < 0.001$, ns, no significance.

Supplementary Figure 2. HK2 expression profile affects the survival of lung cancer (a) Kaplan-Meier curves illustrating overall survival. (b) Kaplan-Meier curves illustrating progression-free survival.

Supplementary Figure 1

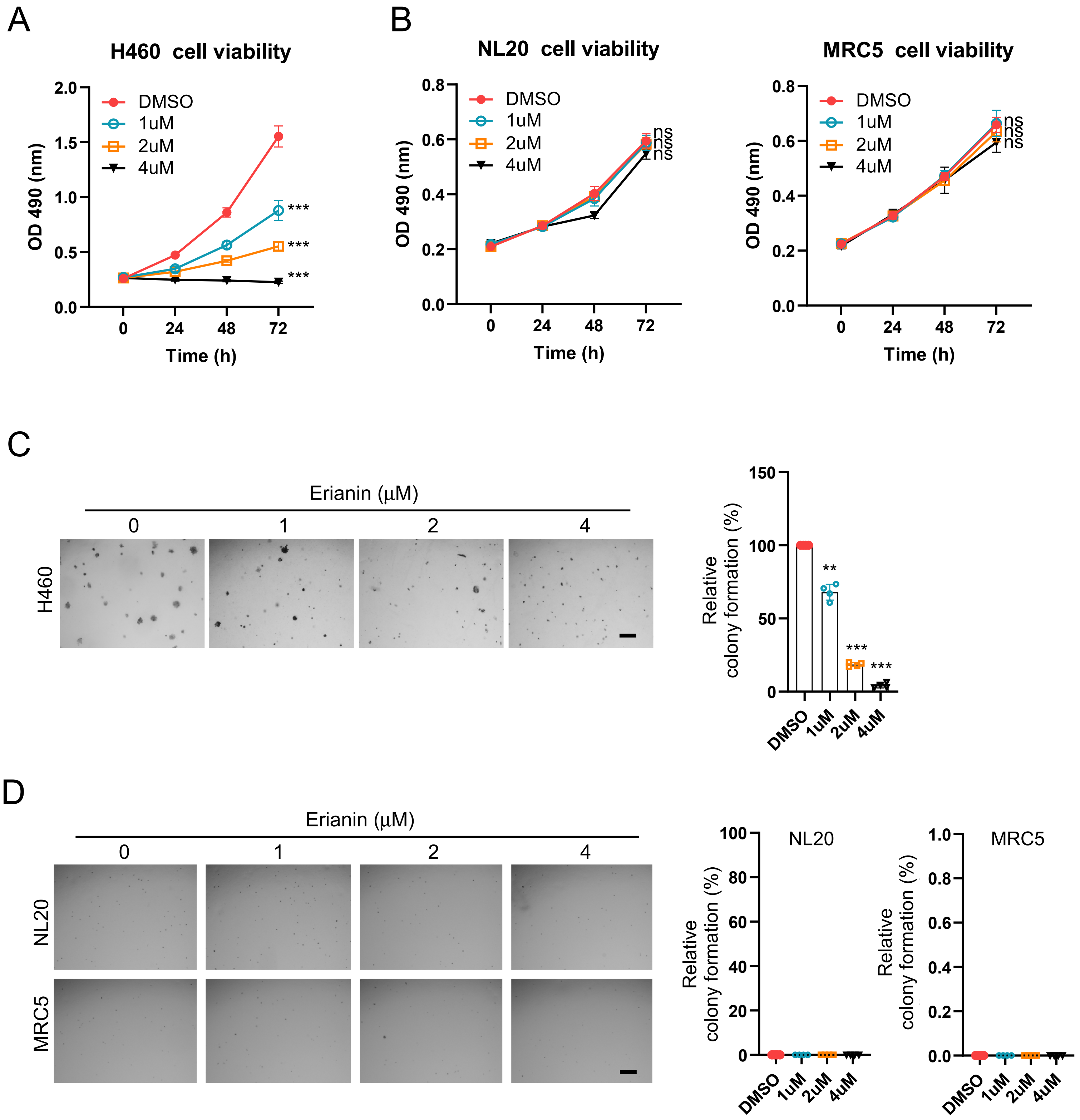

Supplementary Figure 2

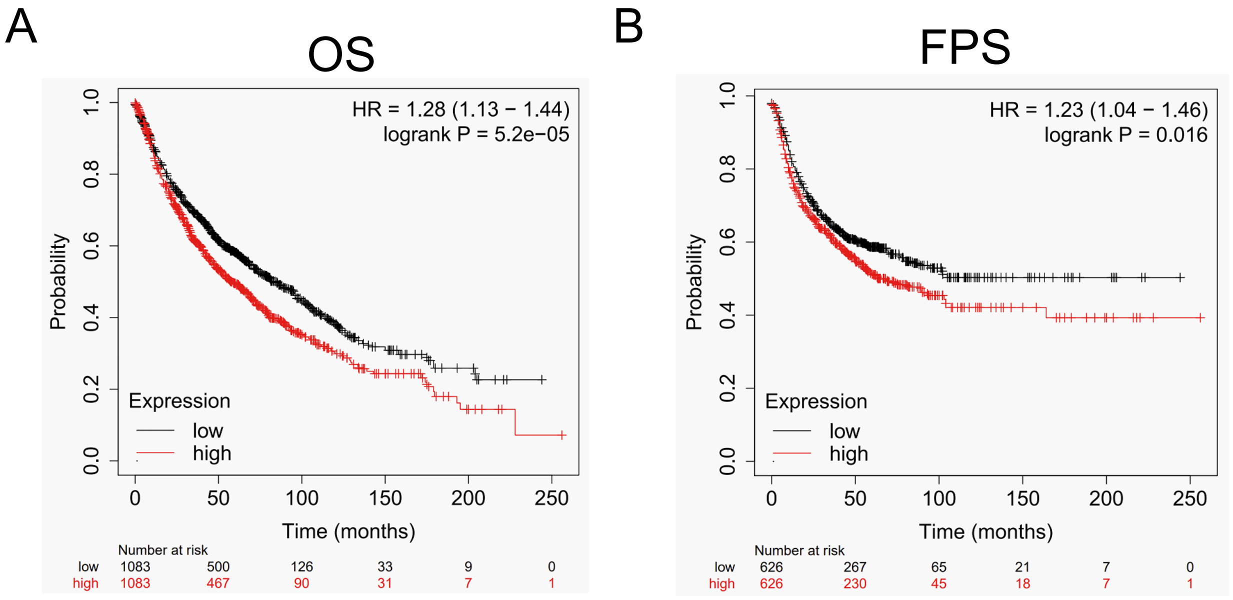

Supplement: Supplementary file 1 — Supplementary figures. [file jcav15p2380s1.pdf]
